# Stoichiometric flexibility in diverse aquatic heterotrophic bacteria is coupled to differences in cellular phosphorus quotas

**DOI:** 10.3389/fmicb.2015.00159

**Published:** 2015-02-27

**Authors:** Casey M. Godwin, James B. Cotner

**Affiliations:** Department of Ecology, Evolution, and Behavior, University of MinnesotaSaint Paul, MN, USA

**Keywords:** aquatic heterotrophic bacteria, phosphorus content, ecological stoichiometry, chemostats, lakes, element quotas, cell morphometry

## Abstract

It is frequently presumed that heterotrophic bacteria from aquatic environments have low carbon (C) content, high phosphorus (P) content, and maintain homeostasis at low C:P in their biomass. Dissolved and particulate organic matter from primary producers in terrestrial and aquatic environments typically has high C:P ratios, suggesting that heterotrophic bacteria consuming this resource experience stoichiometric imbalance in C and P. The strength of elemental homeostasis is important for understanding how heterotrophic bacteria couple C and P cycles in response to environmental change, yet these generalizations are based upon data from only a few species that might not represent the physiology of bacteria in freshwaters. However, recent research has indicated that some strains of bacteria isolated from freshwaters have flexible C:P stoichiometry and can acclimate to changes in resource C:P. Although it is apparent that strains differ in their biomass C:P and flexibility, the basis for these characteristics has not been explained. We evaluated biomass C:P homeostasis in 24 strains of bacteria isolated from temperate lakes using a uniform relative growth rate in chemostats. Overall, the strains exhibited a range of homeostatic regulation from strong homeostasis to highly flexible biomass stoichiometry, but strains that were isolated using P-rich media formulations were more homeostatic than strains isolated using P-poor media. Strains exhibiting homeostatic biomass C:P had high cellular C and P content and showed little morphological change between C and P limitation. In contrast, stoichiometrically flexible strains had low P quotas and increased their C quotas and cell size under P limitation. Because stoichiometric flexibility is closely coupled to absolute P content in bacteria, anthropogenic inputs of P could lead to prevalence of more homeostatic bacteria, reducing the ability of natural assemblages to buffer changes in the availability of P and organic C.

## Introduction

Heterotrophic bacteria couple multiple biogeochemical cycles within terrestrial and aquatic ecosystems (Azam, 1998; Cole, 1999; Schlesinger et al., 2011) and experience imbalance between the chemical composition of their biomass and the chemical composition of their resources. Elemental imbalances and nutrient limitation are not absolute, but rather, they occur relative to the availability of other resources and the physiological requirements of the organism. Adaptations for dealing with resource limitation include reducing growth rate and/or metabolic activity, increasing consumption or uptake rates, minimizing resource loss rates, and minimizing the quantity of the resource required within biomass. Although all organisms exhibit at least one of these mechanisms, flexible biomass composition is particularly important because realized growth rate and resource acquisition rates are coupled indirectly by internal nutrient concentrations (Droop, 1973; van den Berg, 2001). In their role as “gatekeepers” of nutrients within aquatic ecosystems (Kirchman, 1994), the nutrient content and, stoichiometry of bacterial biomass affects the rates at which bacterial communities can remineralize or sequester carbon (C), nitrogen (N), and phosphorus (P) when the supply of these elements is unbalanced relative to their demands (Goldman et al., 1987).

Stoichiometric homeostasis describes how organisms maintain or alter their biomass element composition in response to resource imbalance and environmental change. The strength of stoichiometric homeostasis for C, N, and P differs among major phylogenetic groups (Persson et al., 2010), but there is substantial variation within each group in both the strength of regulation and the range of biomass stoichiometry. Generalizations about the strength of elemental homeostasis within groups of taxa are common in the field of ecological stoichiometry and allow reduction of complex physiological mechanisms to a more tractable mass balance problem. Although such simplifications enable modeling of resource limitation, elemental imbalance, and nutrient regeneration within an assemblage, the biomass stoichiometry and strength of homeostatic regulation for entire trophic levels has been characterized using data from only a few species or strains and might not represent the physiologies present within natural assemblages.

Stoichiometric regulation in bacteria was initially examined using *Escherichia coli*, which exhibited strong homeostasis in biomass C:N:P across two orders of magnitude in P supply (Makino et al., 2003). In a subsequent study, an assemblage of bacteria cultured from a temperate freshwater lake was non-homeostatic, suggesting that assemblages could exhibit non-homeostasis as the result of shifts in relative abundance of strains driven by nutrient availability (Makino and Cotner, 2004). Indeed, assemblages of bacteria from multiple lakes exhibit non-homeostasis and this is partly attributable to selection for different stoichiometric strategies (Godwin and Cotner, 2014). Recent work with bacterial isolates (Scott et al., 2012) has demonstrated that strains encompass a wider range of homeostatic regulation than was presumed, but none of the strains exhibited the strong homeostasis or high growth rates characteristic of *E. coli*. Also, assemblages of bacteria subject to ecological selection at high P availability (low C:P in resources) can exhibit strong homeostasis (Godwin and Cotner, 2014), indicating that homeostatic strains are likely present within natural assemblages. Despite these recent advances, the strength of stoichiometric homeostasis present within assemblages remains poorly characterized.

Just as the ratio of C:N:P within biomass has implications for competition and coexistence within local assemblages (Andersen et al., 2004; Moe et al., 2005), variability in the strength of stoichiometric regulation among related taxa is key to understanding species-level interactions in environments where resource stoichiometry varies across space or time (Jeyasingh et al., 2009; Hood and Sterner, 2010). Due to experimental constraints, measurements of stoichiometric homeostasis are seldom performed for more than a few taxa in a single study. Combining data on stoichiometric regulation from multiple studies introduces unrecognized effects of experimental design and culture conditions, potentially masking important patterns. However, determining the ranges of elemental content and stoichiometric homeostasis within a functional or phylogenetic group is essential to understanding how assemblages regulate their stoichiometry in natural ecosystems.

One problem in studying stoichiometric homeostasis of bacteria from natural environments is that few strains are readily culturable (Colwell and Grimes, 2000) and there may be bias if the medium used for isolation selects for specific physiologies. Specifically, studies of stoichiometric homeostasis in bacteria have been restricted to a small number of strains that were isolated using nutrient-rich media. Bacteria that readily colonize nutrient-rich media often exhibit high growth rates (Staley and Konopka, 1985) and rapidly growing organisms have high P content and P requirements due to the abundance of P-rich ribosomes required at high growth rates (Elser et al., 2000, 2003). Therefore, strains isolated using nutrient-rich media could have different P physiology and stoichiometry than other strains in an assemblage. We hypothesized that P-rich media formulations select for strains with higher P content and more homeostatic physiology than bacteria isolated using dilute P-poor media. We also propose that this bias due to isolation medium has contributed to an under-representation the range of stoichiometric strategies present in natural assemblages.

We sought to answer two questions: (1) Does the medium used for isolation select for different stoichiometric strategies? and (2) Do quotas of C and P differ in systematic ways among homeostatic strains and those with flexible stoichiometry? To address these questions, we characterized growth rates, stoichiometric regulation of C:N:P_biomass_, cell quotas, and morphometry in 24 strains of bacteria isolated from lakes using multiple isolation methods and culture media. Both within and among assemblages, the isolates exhibited a range of element content and degree of stoichiometric regulation, from non-homeostatic strains with low P quotas to strongly homeostatic strains with high P content.

## Materials and methods

### Sample collection and processing

We isolated bacteria from Lake Itasca and Long Lake, both located in or adjacent to the Itasca State Park, Clearwater County, Minnesota, USA. Lake Itasca is moderately productive (7.0 μg L^−1^ chlorophyll-a and 1.07 μmoles L^−1^ total dissolved phosphorus), has a maximum depth of 12 m, and a surface area of 431 hectares. Long Lake is less productive (3.1 μg L^−1^ chlorophyll-a and 0.25 μmoles L^−1^ total dissolved phosphorus), has a maximum depth of 24 m, and a surface area of 64 hectares. Water samples were collected from the upper mixed layer of each lake during the spring using acid-soaked and sterilized polyethylene bottles. Samples were processed within 1 h of collection. The bacteria-sized fraction was separated by filtration through a sterile Whatman GF/B filter (Hall et al., 2009). Cell-free lake water was prepared by filtering the lake water twice using a 0.22 μm pore-size sterile filter (Fisher SteriTop).

### Bacterial isolation and culture methods

#### Dilution isolation and MPN method

To quantify the number of culturable cells obtained using each medium treatment, we performed dilution to extinction isolation (Schut et al., 1993; Page et al., 2004) and most probable number (MPN) assays (Klee, 1993) using the bacterial-sized fraction from each lake. To detect growth of cells, resazurin was added to a final concentration of 20 μmoles L^−1^ in both the inoculum water and the sterile media. Resazurin becomes highly fluorescent when it is reduced by bacterial respiration (Nix and Daykin, 1992; Haines et al., 1996). We diluted the inocula into four media treatments: cell-free lake water, a complex medium (Difco Nutrient Broth, 8 g L^−1^), a defined medium with high phosphorus, and a defined medium with low phosphorus. The composition of the nutrient broth was 1.39 mmoles P L^−1^ (total phosphorus), 1.38 mmoles P L^−1^ soluble reactive phosphorus, and 274 mmoles C L^−1^, resulting in a molar C:N:P of 198:67:1. The defined medium was Basal Microbiological Medium (BMM), prepared following Tanner (2002) using deionized water, with glucose (23.9 mmoles C L^−1^) as the sole source of carbon. Additional minerals, vitamins, and trace metals were supplied at concentrations described in Tanner (2002) and the medium was buffered between pH 7.2 and 7.4 using 11 mmoles L^−1^ 3-(N-Morpholino)propanesulfonic acid (MOPS). Phosphorus was added as potassium phosphate at two levels to create molar C:P of 100:1 (239 μmoles-P L^−1^) and 100,000:1 (0.239 μmoles-P L^−1^). The nutrient broth and BMM medium with C:P of 100:1 are categorized as P-rich media and the cell-free lake water and BMM with C:P of 100,000:1 are categorized as P-poor media.

**Figure 1:**
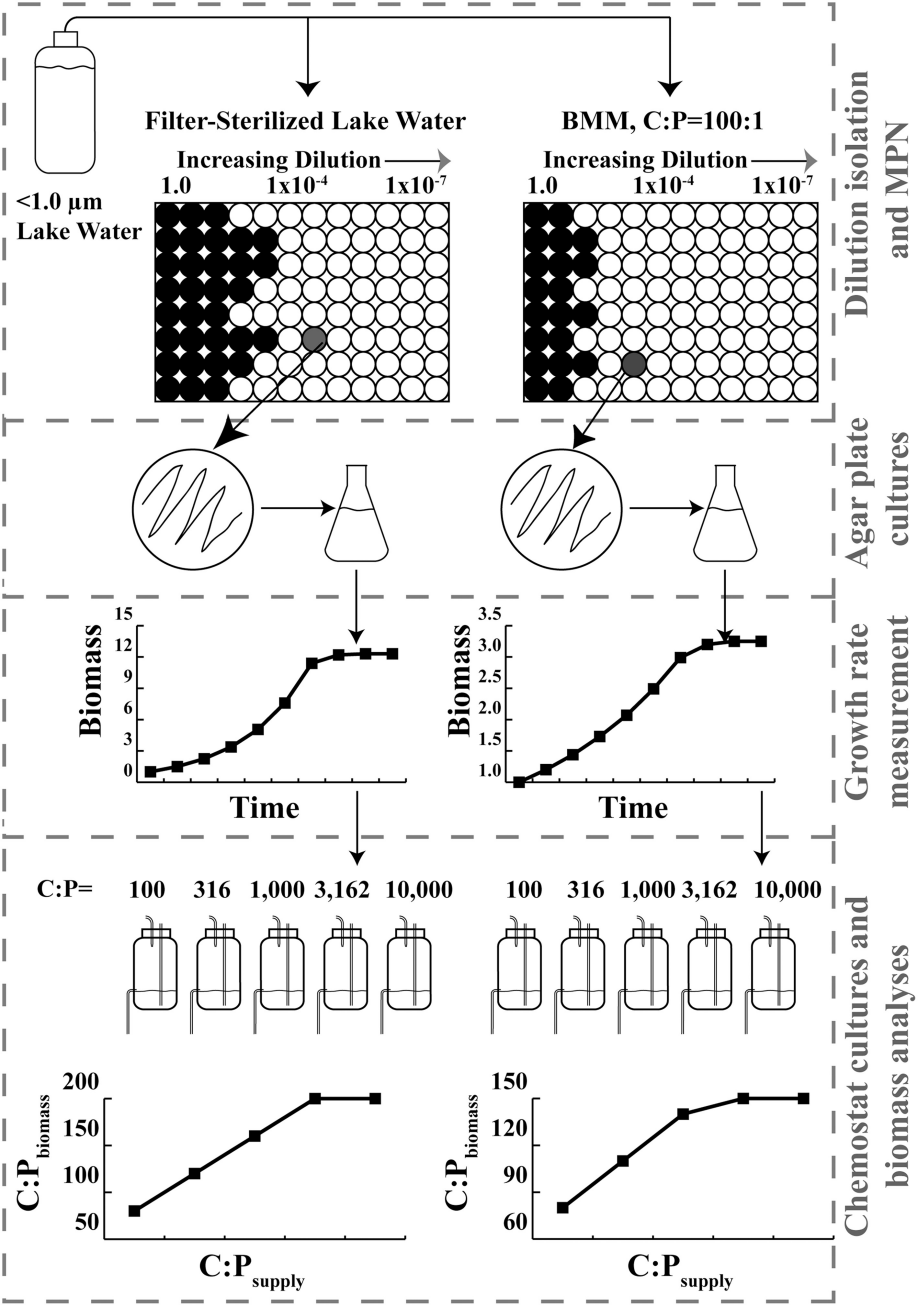
**Schematic diagram of isolation, growth rate measurement, chemostat culture, and results for two different isolates**. Dashed boxes denote Materials and Methods subsections.

Dilution cultures were performed in black 96-well microtiter plates (Nunc) at 11 dilutions between 1 and 2.39 × 10^−7^ (total culture volumes of 170 μL) relative to lake water (Figure 1). Sixty replicate dilution series were performed for each lake in the BMM 100:1 and BMM 100,000:1 treatments. One hundred and eighty replicate dilution series were performed for each lake in the cell-free lake water and nutrient broth treatments. Each microtiter plate contained eight control wells of medium without any inoculum. The plates were sealed with sterile transparent film (Excel Scientific, ThinSeal) and incubated at 20°C in the dark. Fluorescence of the dilution plates was monitored using a using a Fluoromax 3 spectrofluorometer with a MicroMax 384 plate reader (Horbia Jobin Yvon). Fluorescence was measured using excitation at 560 nm and emission at 585 nm, both with 5 nm band pass slit widths. Fluorescence in each well was averaged for 1 s and the plates were returned to the dark incubator between readings. The fluorescence of the plates was measured seven times between 3 and 38 days after inoculation.

After 18 and 38 days, we identified the wells with positive growth as those where the slope of fluorescence vs. time and the absolute fluorescence were both greater than the 90% upper confidence interval for the slope of the control wells for each plate. We computed the MPN estimates and confidence intervals for each lake and medium type following Jarvis et al. (2010). To obtain cultures with a high probability of being axenic, we harvested wells at the highest dilution where the next lowest dilution did not show detectable growth. The contents of these wells were diluted again into the same medium type with resazurin and the fluorescence was monitored for 19 days. Following the second dilution of the potential isolates, we harvested the highest dilution with detectable growth and plated the cultures onto agar made with the same medium. Distinct colony morphologies were preserved as described below.

#### Agar plate cultures

We diluted the bacterial-sized fraction from each lake into cell-free lake water (dilution 1 – 1000×) and plated 100 μL onto agar plates. The plates were prepared using 15 g agar L^−1^ (Fluka number 05038) and each of the following medium formulations: nutrient broth (prepared as above), BMM at C:P of 100:1, and BMM at C:P of 100,000:1. We analyzed the phosphorus content of the agar and it contributed less than 0.263 μmoles P L^−1^ as soluble reactive phosphorus and less than 0.97 μmoles P L^−1^ to the finished media. Five replicate plates were used for each dilution of nutrient broth and 10 replicate plates were used for the BMM formulations. The plates were incubated at 22–24°C and visible colonies were characterized, enumerated, and harvested after 6 and 12 days. For each set of replicate plates, single colonies of each distinct morphology were harvested and streaked onto agar plates with the same media. Permanent cultures were established by adding glycerol to liquid cultures of each isolate (final concentration 15% v/v) and freezing the cultures at −70°C (Morrison, 1977).

#### Isolates selection, identity, and growth rates

Isolates for use in the experiments were randomly selected from the list of potential isolates in each combination of medium treatment and lake. Strains that did not exhibit sufficient growth in liquid batch culture were excluded and other candidates were evaluated until each combination was represented by at least one strain. The strains were assigned to taxonomic affiliation using partial 16S rRNA sequences derived using the primers 8F and 1492R, and subsequent alignment against sequence libraries using the Basic Local Alignment Search Tool (BLAST, National Institutes of Health). To ensure that the strains for this study were not biased toward the isolation methods described above or the biogeography of Long Lake and Lake Itasca, we included seven additional strains provided by Stuart Jones (University of Notre Dame). The strains were isolated from lakes in Indiana and Michigan (Livermore et al., 2014). A strain of *Polynucleobacter necessarius* (Pnec), was obtained from the DSMZ collection (Leibniz Institute, Germany). The source, isolation conditions, and taxonomic affiliation of all of the study strains are given in Table 1.

**Table 1:** **Source and taxonomic affiliation of the strains used for this study**.

**Strain ID**	**Taxonomic affiliation**	**Source**	**Isolation method**
D111	*Betaproteobacteria, Hylemonella*	Lake Itasca, MN	BMM 100:1
D201	*Betaproteobacteria, Hylemonella*	Long Lake, MN	BMM 100:1
D206	*Alphaproteobacteria, Brevundimonas*	Long Lake, MN	BMM 100:1
D301	*Alphaproteobacteria, Rhizobium*	Long Lake, MN	BMM 100,000:1
D304	*Alphaproteobacteria, Rhizobium*	Long Lake, MN	BMM 100,000:1
D611	*Alphaproteobacteria, Brevundimonas*	Long Lake, MN	Sterile lake water
D703	*Alphaproteobacteria, Brevundimonas*	Long Lake, MN	Sterile lake water
D712	*Alphaproteobacteria, Brevundimonas*	Long Lake, MN	Sterile lake water
D801	*Alphaproteobacteria, Brevundimonas*	Long Lake, MN	Sterile lake water
D905	*Alphaproteobacteria, Brevundimonas*	Long Lake, MN	Sterile lake water
D909	*Alphaproteobacteria, Brevundimonas*	Lake Itasca, MN	Nutrient broth
D1207	*Betaproteobacteria, Achromobacter*	Long Lake, MN	Nutrient broth
D1303	*Betaproteobacteria, Achromobacter*	Long Lake, MN	Nutrient broth
P026	Unique partial sequence	Long Lake, MN	BMM 100,000:1
P045	Alphaproteobacteria, Agrobacterium	Long Lake, MN	Nutrient broth
P078	Actinobacteria, Microbacterium	Lake Itasca, MN	Nutrient broth
P089	Not sequenced	Lake Itasca, MN	Nutrient broth
UND-FW12	*Betaproteobacteria, betII (Pnec)*	Pleasant Lake, IN	WC minimal medium
UND-L13	*Betaproteobacteria*	Little Long Lake, MI	WC minimal medium
UND-L18	*Gammaproteobacteria, gamIV gamIV-A Pseudo A1*	Little Long Lake, MI	WC minimal medium
UND-L41A	*Alphaproteobacteria, alflI Brev*	Little Long Lake, MI	WC minimal medium
UND-Pnec	*Polynucleobacter necessarius*	DSMZ culture collection	–
UND-WG21	*Bacteroidetes, Flavobacteria*	Wintergreen Lake, MI	WC minimal medium
UND-WG36	*Gammaproteobacteria, gamII gamII-A*	Wintergreen Lake, MI	WC minimal medium

Because the absolute and relative growth rates of microorganisms affect their stoichiometry and flexibility (Makino and Cotner, 2004; Hillebrand et al., 2013), we normalized the dilution rate for each strain to its apparent maximum growth rate. The apparent maximum growth rate of each isolate (μ_max_) was measured in BMM medium with high P availability (C:P = 100:1) using batch cultures with a volume of one milliliter. Since these culture conditions are not specific to each isolate, the apparent μ_max_ likely underestimates the actual maximum growth rate. Cultures of each isolate were inoculated from permanent cultures and incubated at 22–24°C on an orbital shaker. At each time point, two replicates of each isolate were rapidly frozen using liquid nitrogen and were stored at −70°C until analysis. The population growth rate of bacteria in each culture was determined from the change in the concentration of double-stranded DNA using the PicoGreen reagent (Invitrogen Quant-It PicoGreen Kit) and fluorescence measurement (Tranvik, 1997; Cotner et al., 2001). Poor sensitivity was observed when the PicoGreen reagent was added directly to cells growing in medium. Sensitivity was improved substantially by extracting the DNA prior to quantification. After thawing the cultures, 125 μL of extraction buffer (29.1 mmoles L^−1^ sodium lauryl sarcosine, 54 mmoles L^−1^ tris(hydroxymethyl) aminomethane, and 5.4 mmoles L^−1^ ethylene(diamine)tetraacetic acid at pH 8.0) was added to the samples (Gorokhova and Kyle, 2002) and the samples were incubated at 22–24°C on a rotary shaker for 1 h. DNA standards (Invitrogen) were prepared by dilution into TE buffer (10 mmoles L^−1^ tris(hydroxymethyl) aminomethane and 1 mmoles L^−1^ ethylene(diamine)tetraacetic acid at pH 8.0). The PicoGreen reagent was diluted 1:470 in extraction buffer and 150 μL was added to each sample, followed by mixing. The samples were incubated in the dark for at least 10 min and transferred to 1 cm polymethylmethacrylate cuvettes (VWR Scientific). The fluorescence was measured using excitation of 500 nm and emission at 523 nm (5 nm band pass slit widths) using a Fluoromax 3 fluorometer (Horbia Jobin Yvon). Fluorescence values were averaged over 1 s. The working range of the assay was 50 pg to 200 ng DNA mL^−1^. Growth rates were estimated from cultures where the DNA concentration increased exponentially (log-linear *R*^2^ > 0.9) for at least three successive time points and the DNA concentration was less than 40 ng mL^−1^. Based upon a range of 3–20 fg DNA celL^−1^ in cultured cells (Cotner et al., 2001; Makino and Cotner, 2004), the approximate cell densities used for growth rates were between 3.3 × 10^4^ and 1×10^7^ cells mL^−1^. The growth rate estimates from replicate cultures (μ_max_) were within 10%.

### Chemostat cultures and biomass analyses

#### Chemostat cultures

Each isolate was cultured in 100 mL polypropylene chemostats diluted at 33% of μ_max_(Figure 1). The P content of the BMM formulation was manipulated to achieve molar C:P ratios of 100, 316, 1000, 3162, and 10,000:1 (2.4–239 μmoles P L^−1^). Batch cultures for each treatment were inoculated with aliquots of the permanent cultures. After the batch cultures reached optical density at 600 nm greater than 0.05 cm^−1^, 5 mL of the batch cultures was used to inoculate duplicate chemostats at each level of C:P_supply_. Chemostats were maintained at 20°C in darkness, aerated, and mixed with 0.2 μm-filtered air, and harvested after 9 complete turnover times.

#### Dry mass and elemental content

Samples of biomass were collected from each chemostat using Whatman GF/F filters that were combusted at 450°C. Three replicate filters were stored in a desiccator until weighing to the nearest 0.1 μg for determination of dry mass. The filters were rinsed with 10% hydrochloric acid and then with deionized water prior to harvesting the cells under low vacuum (<100 mm Hg). The filter samples were rinsed with deionized water to remove excess media, and stored at −20°C until analysis. Filters for dry mass were dried at 60°C until constant mass (7 days) and weighed again, and the blank-corrected difference was used as dry biomass. Blank filters prepared with deionized water and filters from sterile chemostats showed no significant difference in biomass accumulation (Godwin and Cotner in review). One filter from each chemostat was randomly selected for direct measurement of C and N content using a CHN analyzer (Perkin-Elmer 2400CHN) with acetanilide (Elemental Microanalysis Ltd.) as a primary standard and a zooplankton-derived recovery standard. Three filter samples from each chemostat were analyzed for bacterial P content. Following digestion in 25 g L^−1^ potassium persulfate at 121°C for 30 min (APHA, 1995), the phosphorus content was determined using the ascorbic acid molybdenum method. Spinach leaf reference material (NIST) was used as a recovery standard for all phosphorus analyses (mean recovery 94.4%). The apparent yields of C and P were computed as the proportion of C and P available in supply that was recovered as biomass from the chemostats.

#### Cell abundance and morphometry

Aliquots of the chemostat cultures were preserved with 0.2 μm-filtered formaldehyde (3.7% by volume) and stored at 4°C. One sample from each chemostat was prepared for microscopic enumeration by dilution in sodium pyrophosphate and sonication (Velji and Albright, 1993). Each sample was stained with acridine orange, filtered onto black polycarbonate membrane filters (Nucleopore, 0.2 μm pore size), washed with cell-free deionized water, and mounted to slides for microscopy (Hobbie et al., 1977). Cell counts and morphometry measurements were performed at 1000× magnification using an Olympus BX40 microscope. For cell counts, at least 10 fields and 300 cells on each filter were enumerated manually. Photomicrographs were obtained using a digital camera (Spot 2, Diagnostic Instruments) at 1000× magnification. Cell dimensions (length, width, planar area, and planar perimeter) were measured for at least 100 cells from each chemostat using image analysis software (Image Pro Plus, Media Cybernetics). Cell shape was measured as cylinders capped with two hemispheres (Hillebrand et al., 1999). Due to the high proportion of curved cells, cell dimensions were calculated using the planar area and perimeter, rather than the box length and width. The equations used for estimating cell length, width, surface area, and volume from planar area and perimeter are given in the Supplementary Materials.

### Statistical analyses

The mean blank-corrected measurements for each chemostat were used to calculate molar ratios of elements (C:P_biomass_, N:P_biomass_, and C:N_biomass_). The elemental quotas of the cells (P celL^−1^ and P relative to dry mass) were computed as the mean particulate P divided by the cell density. In several strains, quotas of each C, N, and P increased under P limitation (C:P_supply_ of 10,000:1) relative to P sufficiency (C:P_supply_ of 100:1). For these strains, the C:N:P of the added biomass was calculated as the molar ratio of the increase for each element. For each strain, the strength of stoichiometric regulation was assessed using segmented linear regressions (Kim et al., 2004) of log_10_ C:P_biomass_ against log_10_ C:P_supply_ (Sterner and Elser, 2002). The break point was selected by iteratively bisecting the data series at each level of C:P_supply_ from 316:1 to 10,000:1 and performing standard linear regression on the lower (flexible) and upper (homeostatic) ranges. The breakpoint was chosen as the level of C:P_supply_ that minimized the total sum of squares for both segments. Strains were separated into three even categories (stoichiometric categories) based upon the degree of flexibility in C:P_biomass_ observed in the chemostat cultures.

Morphometric data were log_10_-transformed prior to analysis to meet assumptions of approximate normality and homogeneity of variances (Sokal and Rohlf, 1995). Biomass elemental content, morphometric data, and yields were analyzed by analysis of covariance (ANCOVA) tests, using stoichiometric category as a fixed effect and C:P_supply_ as a quantitative treatment. When a significant interaction was observed, separate One-Way analysis of variance (ANOVA) tests were performed for each level of C:P_supply_. Pairwise differences in the One-Way ANOVA analyses were evaluated using Tukey's Honest Significant Difference tests, with a significance cutoff of *p* < 0.05. To determine the effect of isolation medium on homeostatic classification of the isolates, the strains from Lake Itasca and Long Lake were grouped into those isolated using P-rich media (nutrient broth and BMM with C:P of 100:1) and P-poor media (sterile lake water and BMM with C:P of 100,000:1). The proportions of isolates belonging to each stoichiometric category were compared for P-rich and P-poor media using a chi-squared test with the null hypothesis that the categories are evenly distributed among medium types (Sokal and Rohlf, 1995).

## Results

### Cultivation and medium formulations

**Figure 2:**
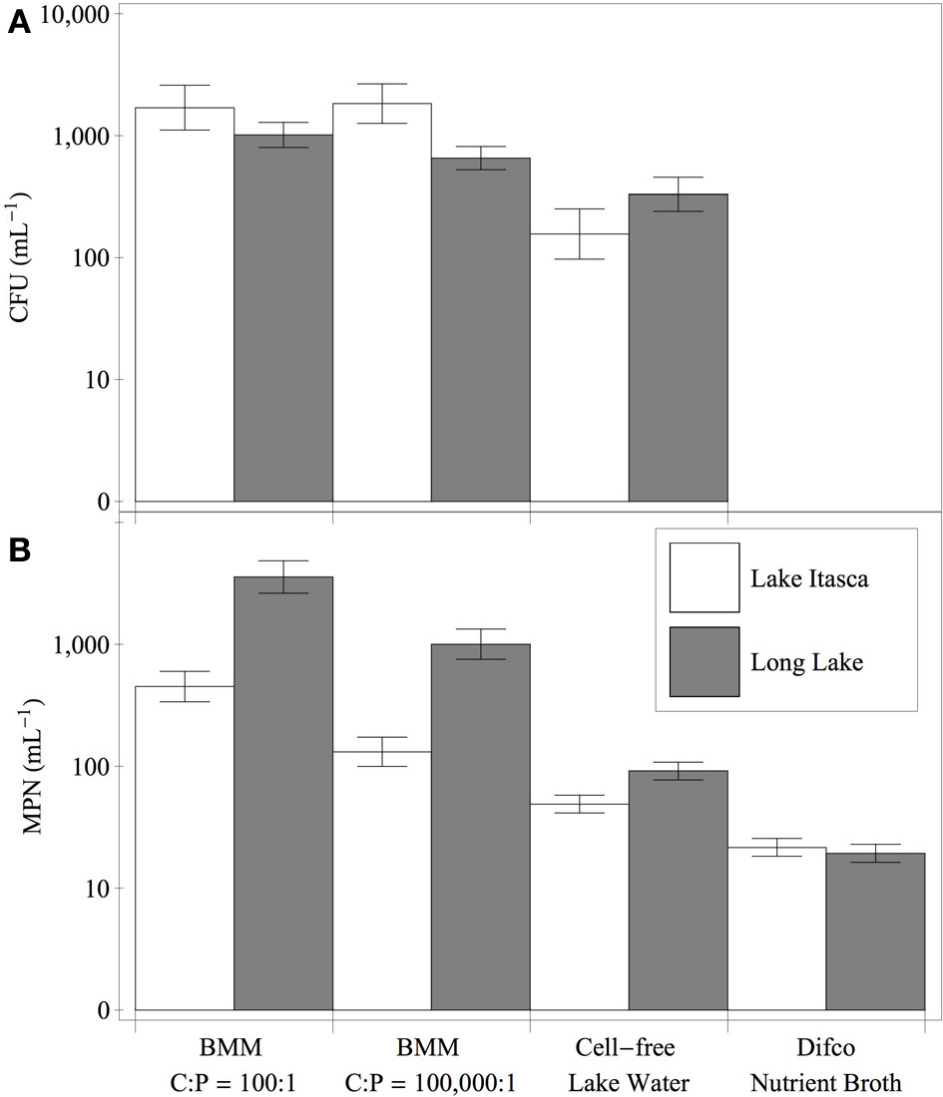
**Cultivation yields of bacteria from Long Lake and Lake Itasca on P-rich and P-poor media formulations**. Panels show colony-forming units **(A)** and most probable number estimates **(B)** for water samples inoculated into different medium treatments. Error bars denote the 95% confidence intervals for the estimates.

The bacterial assemblages diluted into a defined medium (BMM) with C:P of 100:1 showed significantly higher MPN estimates than the samples diluted into cell-free lake water or nutrient broth (Figure 2). The MPN estimates for Long Lake were higher than those for Lake Itasca in all medium treatments except for the nutrient broth. Samples plated onto solid media showed a similar pattern of reduced counts for nutrient broth compared to both BMM treatments (Figure 2). The ANCOVA for CFU mL^−1^ revealed significant effects of medium treatment (*p* < 0.003), and lake (*p* < 0.03), but no interaction (*p* > 0.10). *Post-hoc* tests indicated that the nutrient broth had significantly lower CFU mL^−1^ than the BMM medium formulations (Tukey HSD, *p* < 0.05). The growth rates of the isolates ranged from 0.07 to 0.43 h^−1^ (Table 2). For the isolates from Lake Itasca and Long Lake (MN), there was no effect of isolation medium richness on growth rate (Wilcoxon two-sample test, *p* > 0.05).

**Table 2:** **Growth rate, biomass stoichiometry, and strength of homeostasis for isolates**.

**Isolate**	**μ_max_ (h^−1^)**	**C:P_biomass_**	**N:P_biomass_**	**Slope**
D111	0.116	88–132^*^	13–19	0.037
D201	0.153	*68–78*	*25–33*	0.036
D206	0.089	–	–	–
D301	0.142	64–280^*^	15–38^*^	0.926
D304	0.089	75–399^**^	16–43^*^	0.730
D611	0.091	103–374^***^	19–53^***^	0.497
D703	0.091	104–869^***^	19–124^***^	0.570
D712	0.124	87–297^***^	16–44^**^	0.384
D801	0.120	80–421^***^	15–67^***^	0.548
D905	0.112	90–287^***^	17–43^**^	0.480
D909	0.091	91–566^**^	17–78^**^	0.528
D1207	0.086	86–160^*^	19–28	0.079
D1303	0.138	53–116^**^	12–22^*^	0.400
P026	0.112	*62*–*160*	*9*–*22*	0.295
P045	0.219	67–146^**^	15–28^*^	0.464
P078	0.074	*66*–*85*	*15*	0
P089	0.222	*67*–*102*	*15–27*	−0.011
UND-FW12	0.078	80*–*179^***^	18*–*36^***^	0.146
UND-L13	0.342	50–241^*^	9–40^*^	−0.117
UND-L18	0.249	52–222^*^	11–25^***^	0.140
UND-L41A	0.144	100–372^*^	15–40^***^	0.158
UND-Pnec	0.204	*71*–*192*	*17*–*44*	−0.040
UND-WG21	0.097	79–162^***^	16–34^***^	0.350
UND-WG36	0.432	72–190^**^	13–32^***^	0.579

Stoichiometry data are the ranges of mean values for each level of C:P_supply_. The slope is the linear regression below the breakpoint in C:P_supply_. The p-value associated with the One-Way ANOVA of each parameter vs. C:P_supply_ is denoted by

* *p < 0.05*,

** p < 0.01, and

*** *p < 0.001. Italics denote strains where fewer than 10 chemostats were within detection limits for the parameter*.

### Biomass stoichiometry

**Figure 3:**
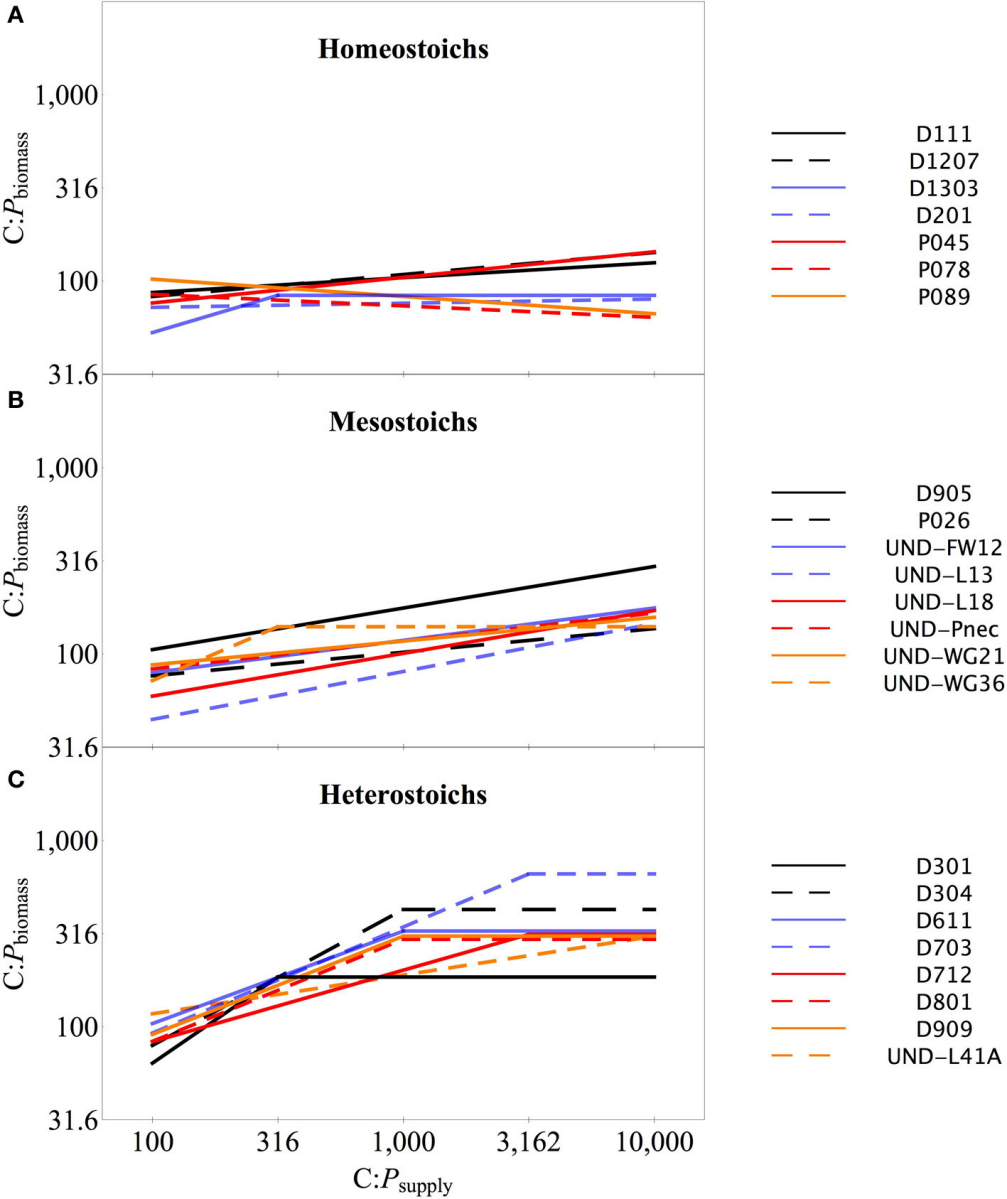
**Biomass C:P stoichiometry across C:P_supply_ for isolates in each category**. Biomass C:P stoichiometry for the isolates in each category: homeostoichs **(A)**, mesostoichs **(B)**, and heterostoichs **(C)**. Lines denote the segmented linear regression as described in the text.

The strains exhibited variable stoichiometry in chemostat cultures, with C:P_biomass_ ranging from 47:1 to 994:1 and N:P_biomass_ ranging from 8.2:1 to 132:1 (Table 2). C:N_biomass_ was less variable, ranging from 2.3:1 to 11:1. ANCOVA tests on C:P_biomass_, N:P_biomass_, and C:N_biomass_ indicated significant effects of strain, C:P_supply_, and an interaction (all *p* < 0.0001). Separate One-Way ANOVA tests for each strain indicated significant effects of C:P_supply_ for a subset of the strains (Table 2). The regression slopes of log C:P_biomass_ vs. log C:P_supply_ (Sterner and Elser, 2002) below the breakpoint ranged from −0.09 to 0.93 (Table 2, Figure 3). The strains were assigned into three arbitrary categories using the lower, middle, and upper third of the range in C:P_biomass_. Homeostoichs exhibited ranges of C:P_biomass_ less than 83, mesostoichs had ranges of C:P_biomass_ from 83 to 210, and heterostoichs had ranges of C:P_biomass_ greater than 210 (Figure 3). For the isolates from Lake Itasca and Long Lake (MN), medium types produced different proportions of homeostoich, mesostoich, and heterostoich strains (chi-squared test, *p* < 0.018). P-rich media formulations produced 6 homeostoichs, 1 mesostoich, and 1 heterostoich and P-poor media yielded 1 homeostoich, 1 mesostoich, and 5 heterostoichs.

**Figure 4:**
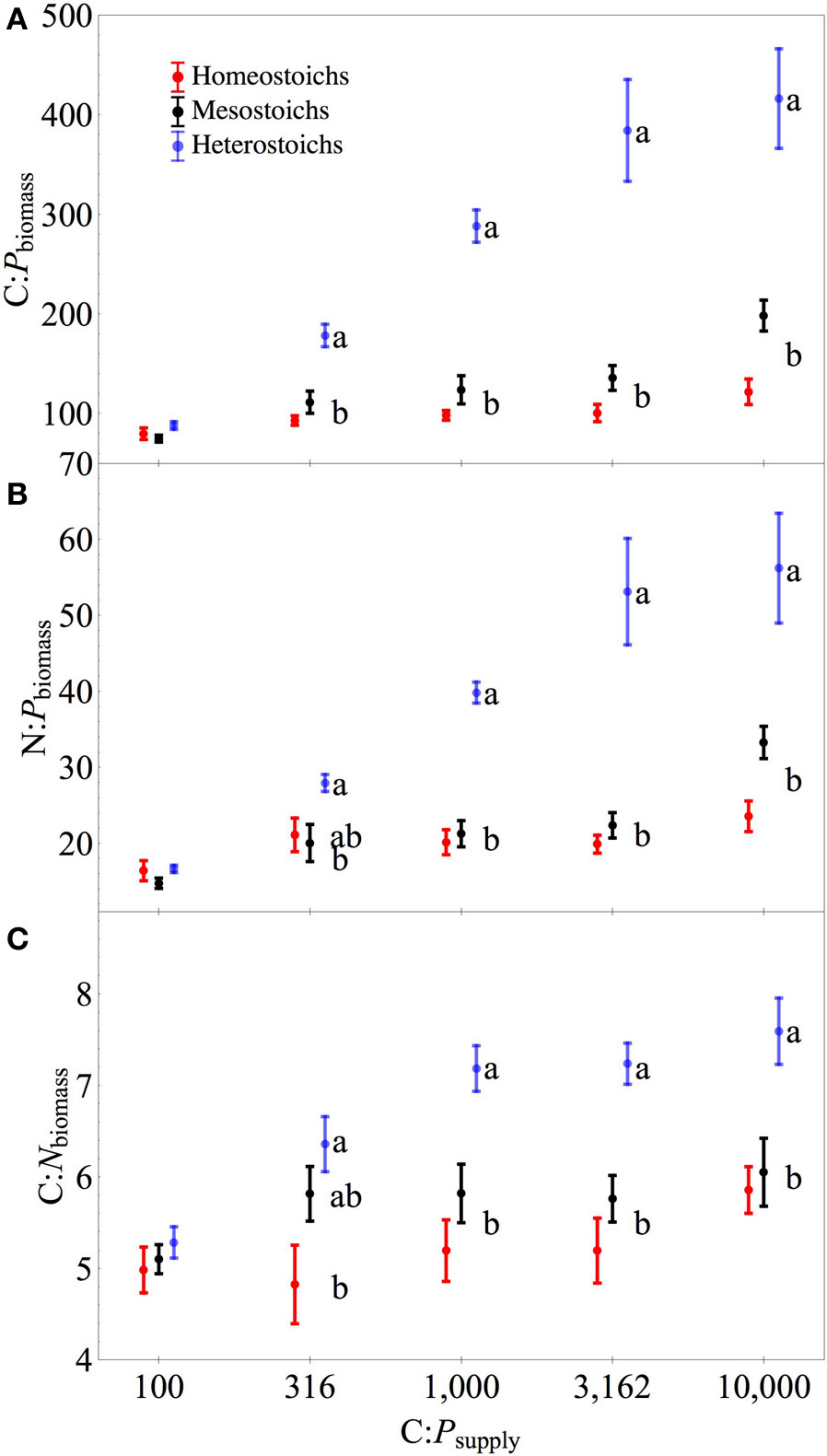
**Biomass stoichiometry of isolates by stoichiometric category**. Separate panels for C:P **(A)**, N:P **(B)**, and C:N **(C)** stoichiometry across C:P_supply_. Error bars denote one standard error of the mean. Lower case letters denote significantly different subsets of the stoichiometric categories at each level of C:P_supply_ (Tukey HSD, *p* < 0.05). Symbols for each category are staggered horizontally at each level of C:P_supply_ to improve clarity.

At C:P_supply_ of 100:1, there were no significant differences in biomass stoichiometry among the stoichiometric categories (all *p* > 0.05), with C:N:P_biomass_ ranging from 52:11:1 to 104:19:1 and a median ratio of 81:16:1. Under P limitation, mean C:N:P_biomass_ for each isolate ranged from 116:21:1 to 869:124:1. Using the stoichiometric categories as groups (Figure 4), C:P_biomass_ and N:P_biomass_ each showed significant effects of C:P_supply_, category, and an interaction (all *p* < 0.0001). All three categories of strains exhibited increased N:P_biomass_ under P limitation (*p* < 0.05). Mean C:N_biomass_ was also affected by C:P_supply_ (*p* < 0.0001), category (*p* < 0.0001), and an interaction (*p* < 0.05). For heterostoichs and mesostoichs, C:N_biomass_ increased under P limitation (*p* < 0.05), but homeostoich C:N_biomass_ did not change (Figure 4). For the isolates from Lake Itasca and Long Lake (MN), P-rich media produced isolates with lower ranges in C:P_biomass_, N:P_biomass_, and C:N_biomass_ (Wilcoxon test, all *p* < 0.01). Minimum C:P_biomass_ showed a negative correlation with μ_max_ (*r*^2^ = 0.24, *p* < 0.02).

**Figure 5:**
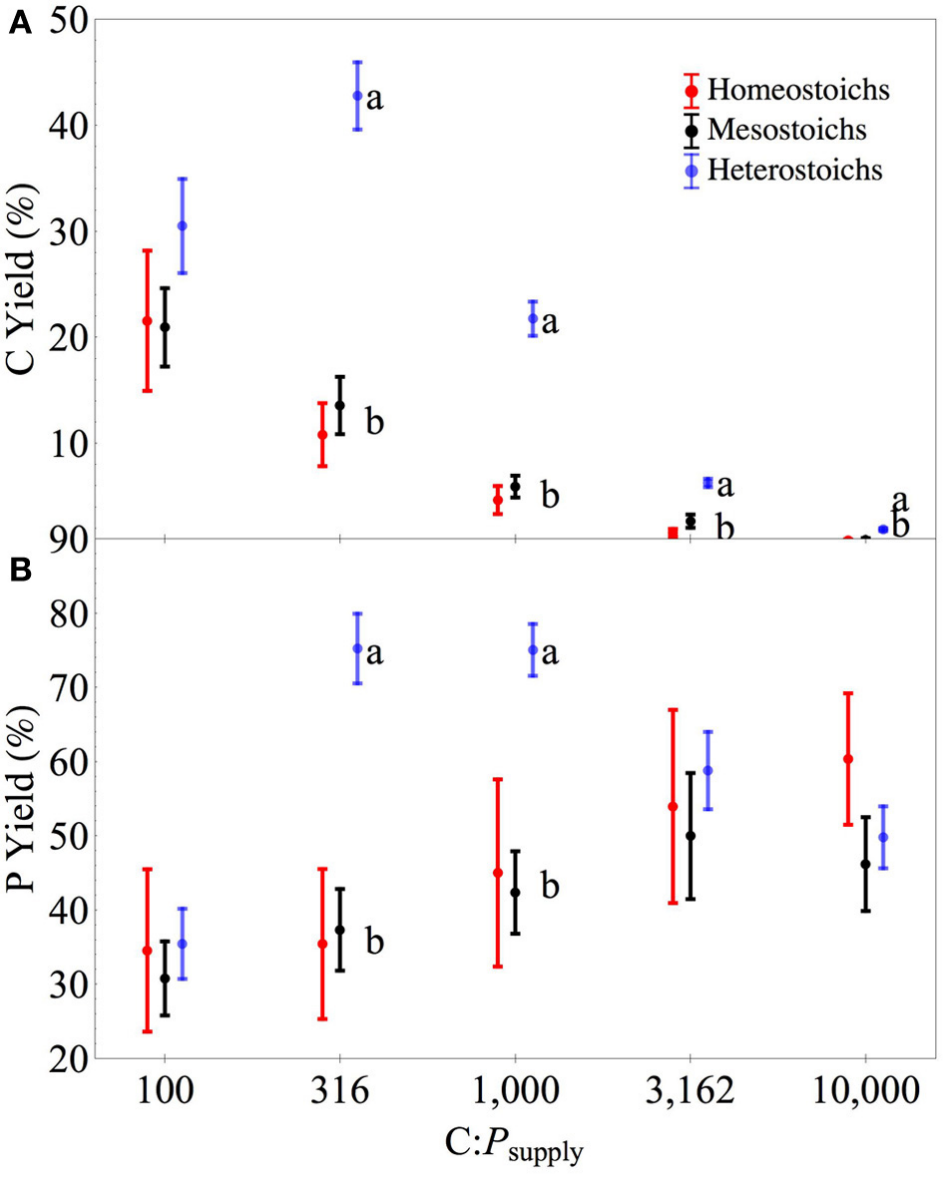
**Carbon and phosphorus quotas of isolates by stoichiometric category**. Separate panels for cellular carbon **(A)** and phosphorus **(B)** quotas across C:P_supply_. Error bars denote one standard error of the mean.

Abundance of the cells in the cultures decreased with increasing C:P_supply_ and the strength of the decrease was proportionally different among the categories (ANCOVA, *p* < 0.0001). At all levels of C:P_supply_, heterostoichs had higher cell abundance than mesostoichs or homeostoichs. As a percentage of available C, biomass C yield was higher in the heterostoich strains compared to the mesostoichs and homeostoichs at all levels of C:P_supply_ above 100:1 (Figure 5, *p* < 0.05). Between C:P_supply_ of 316:1 and 1000:1, heterostoichs also exhibited higher P yield (mean 75%) than the homeostoichs and mesostoichs (35–45%, *p* < 0.05). Although the residual P was not measured in the chemostats, the recovered biomass P was lowest at C:P_supply_ of 10,000:1. Inorganic P was supplied at 2.39 μmoles L^−1^ in the 10,000:1 medium treatment and the mean biomass P was 1.19 μmoles L^−1^ for heterostoichs, 1.44 μmoles L^−1^ for mesostoichs, and 1.103 μmoles for homeostoichs (ANOVA by category *p* < 0.002).

### Cellular C and P content

**Figure 6:**
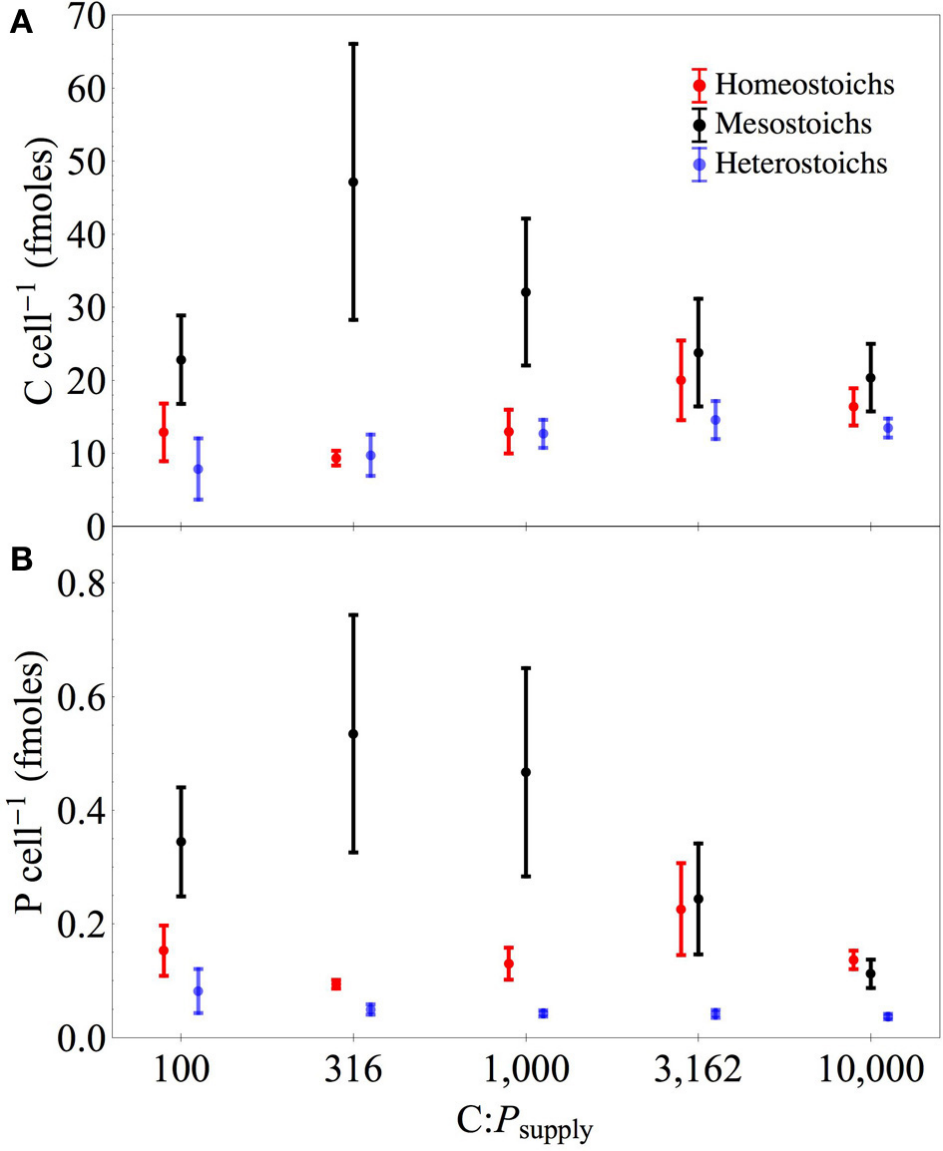
**Carbon and phosphorus yields of isolates by stoichiometric category**. Separate panels for carbon **(A)** and phosphorus **(B)** yields across C:P_supply_. Error bars denote one standard error of the mean.

Phosphorus quotas of the isolates ranged from 0.013 to 1.57 fmoles celL^−1^ and C quotas ranged from 1.04 to 143 fmoles celL^−1^ (Table 3, Figure 6). The ANCOVA tests on cell quotas indicated a significant effect of C:P_supply_ and an interaction between C:P_supply_ and strain (all *p* < 0.0001). One-Way ANOVA tests on C and P quotas indicated significant effects of C:P_supply_ in only a subset of the strains (Table 3). Relative to cell volume, P content ranged from 0.009 to 1.53 fmoles μm^−3^ and C content ranged from 2.9 to 126 fmoles μm^−3^. Under P limitation, P quotas relative to cell volume increased significantly only in one strain, but decreased significantly in eight strains (Table 3).

**Table 3:** **Elemental content of the isolates**.

**Isolate**	**P/dry mass (%)**	**P cell^−1^ (fmoles)**	**C cell^−1^ (fmoles)**	**P volume^−1^ (fmoles μm^−3^)**	**C volume^−1^ (fmoles μm^−3^)**
D111	0.57–1.09	0.191–0.245	17.7–31.1	0.726–0.960	67.7–121.61
D201	*0.17–0.38*	*0.015–0.074*	*1.03–5.79*	*0.054–0.341*	*3.68–26.7*
D206	–	*0.042–0.051*	–	*0.030–0.033*	–
D301	0.38–1.80^**^	0.062–0.075	4.4–17.5^*^	0.052–0.110^*^	7–14.5
D304	0.33–1.74	0.028–0.06^*^	2.1–22.0^**^	0.028–0.039	2.9–12.7^**^
D611	0.36–1.05^***^	0.019–0.031^**^	2.0–11.5^***^	0.042–0.062	6.3–18.8^***^
D703	0.03–0.76^***^	0.013–0.028	1.0–11.6^***^	0.009–0.059	3.6–24.8^*^
D712	0.06–1.25^**^	0.023–0.042	2.1–8.4^***^	0.041–0.09	6.8–14.8^**^
D801	0.11–1.20^***^	0.021–0.029	1.7–12.1^***^	0.037–0.096^**^	7.7–17.6^**^
D905	0.28–1.18^***^	0.026–0.038	2.9–9.6^***^	0.044–0.127^*^	9.2–15
D909	0.11–0.85^***^	0.033–0.084	3.1–37.5^*^	0.048–0.16	13.1–83.5
D1207	0.68–1.42^*^	0.032–0.13^**^	2.6–17.5^***^	0.106–0.493^**^	8.3–54.7^***^
D1303	1.01–2.08^**^	0.076–0.12	4.0–12.5^***^	0.155–0.259	8.2–25^**^
P026	0.32–1.35	*0.143–0.302*	*18.0–21.9*	*0.568–1.264*	*71.7–91.2*
P045	0.29–1.52^***^	0.11–0.23	11.7–27.8^*^	0.11–0.284	12.3–33.7
P078	*0.21–0.31*	*0.071–0.146*	*6.07–9.25*	*0.335–0.603*	*28.5–38.3*
P089	*0.27–1.03*	*0.440–0.768*	*41.19–50.96*	*0.756–0.888*	*58.9–71.8*
UND-FW12	0.24–0.84^**^	0.037–0.088^*^	5.4–9.2	0.124–0.38^*^	17.8–32.5
UND-L13	0.21–1.92^*^	*0.92–1.57*	*73.7–80.8*	*0.892–1.526*	*72.1–85.4*
UND-L18	0.28–1.85**	0.28–1.18	27.6–142.6	0.29–1.031^*^	29.2–125.2
UND-L41A	0.28–1.13^**^	0.024–0.45^**^	4.7–46.5	0.06–1.238^**^	11.5–126.4^*^
UND-Pnec	*0.05–0.36*	*0.118–0.306*	*22.4–24.0*	*0.484–1.32*	*92.3–103.6*
UND-WG21	0.20–1.18^**^	0.039–0.15*	*4.7–12.0*	0.106–0.335*	*12.7–26.6*
UND-WG36	0.25–1.40^***^	0.10–0.17^*^	12.1–21.9	0.173–0.362^*^	25.9–36.5

Data are the ranges of mean values for two chemostats at each level of C:P_supply_. The p-value associated with the one-way ANOVA of each parameter vs. C:P_supply_ is denoted by

* *p < 0.05*,

** p < 0.01, and

*** *p < 0.001. Underlining denotes samples with insufficient replication for ANOVA, italics denote strains where fewer than 10 chemostats were within detection limits for the parameter*.

For the isolates from Lake Itasca and Long Lake (MN), P-poor media produced isolates with lower minimum P quotas (by volume, Wilcoxon test, *p* < 0.03) and lower maximum P quotas (per cell, *p* < 0.02). Isolation medium type did not affect minimum C quotas, but P-poor media produced isolates with significantly lower maximum C quota (by volume, *p* < 0.004). At C:P_supply_ of 100:1, μ_max_ was positively correlated with P quotas (*r*^2^ = 0.37, *p* < 0.003) and carbon quotas (*r*^2^ = 0.30, *p* < 0.008) among the isolates. Relative to dry mass, P content of the isolates ranged from 0.032 to 2.08% and showed significant effects of stoichiometric category and C:P_supply_ (ANCOVA, all *p* < 0.001). Under P limitation, all of the strains exhibited decreased P content relative to dry mass, although this decrease was statistically significant in only 16 of the strains (Table 3).

**Figure 7:**
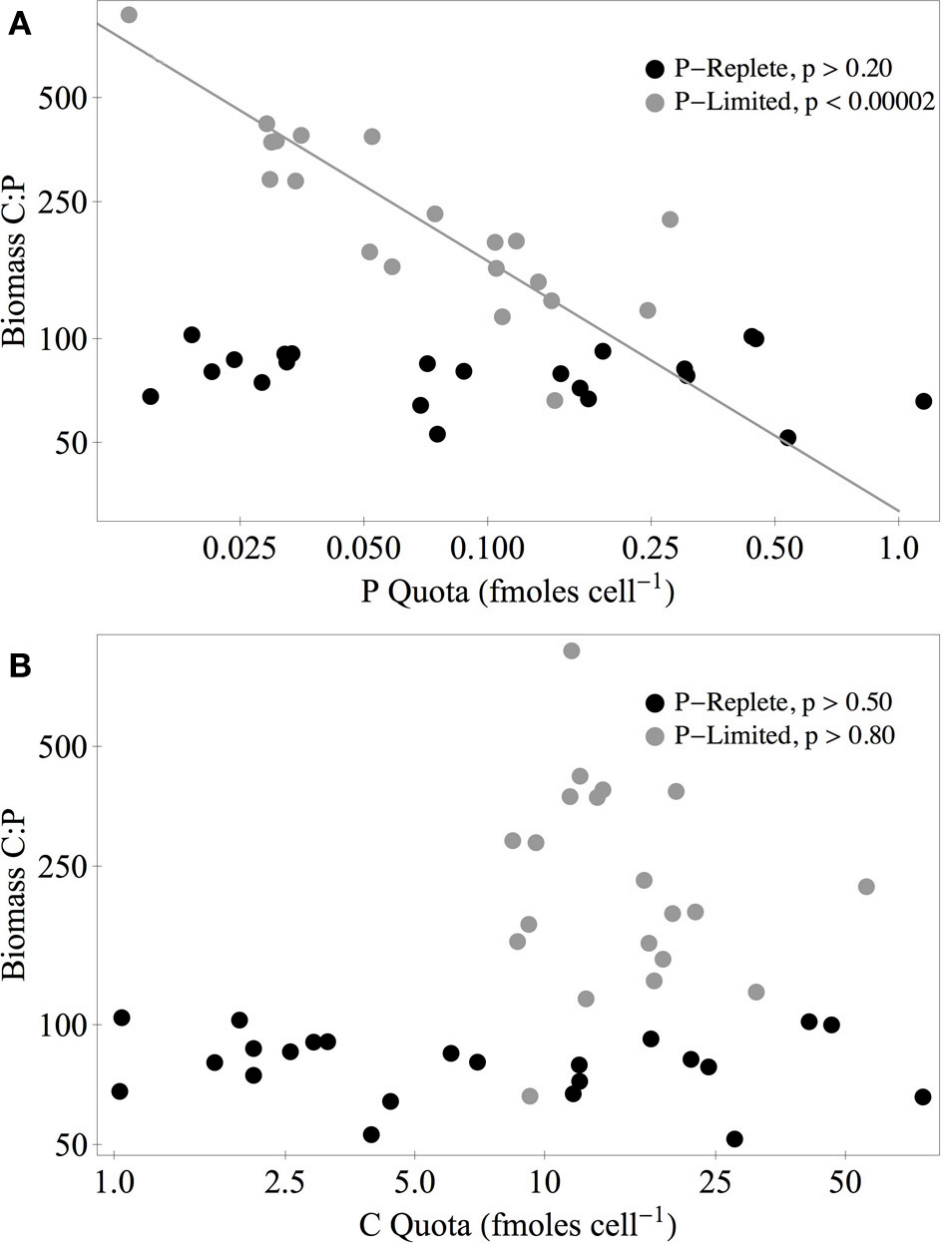
**Biomass C:P vs. P (A) and C quotas (B) of all isolates under P-replete (C:P = 100:1, black circles) and P-limited conditions (C:P = 10,000:1, gray circles)**. The solid gray line represents the standardized major axis regression (Warton et al., 2006) of log-transformed data. *P*-values are from ANOVA tests of the regression slope (H_0_: slope = 0).

Under P-replete conditions, C:P_biomass_ of the isolates was not strongly related to biomass C and P content (Figure 7). Under P limitation, there was a significant negative relationship between C:P_biomass_ and P quota, but there was no relationship with C quota. Of the 11 strains that increased both their absolute C quota and P quota under P limitation, the C:P of the added biomass between C:P_supply_ of 100:1 and 10,000:1 ranged from 205:1 to 5866:1. For the seven heterostoich strains exhibiting increased quotas of C and P under P limitation, the mean C:P of the added biomass was 1964:1. In 13 of the strains, both C and N quotas increased under P limitation and the C:N of added biomass ranged from 5:1 (P045) to 63:1 (UND-WG36). In the 10 strains where both N and P increased under P limitation, N:P of the added biomass ranged from 17:1 to 372:1.

### Cell morphometry

**Figure 8:**
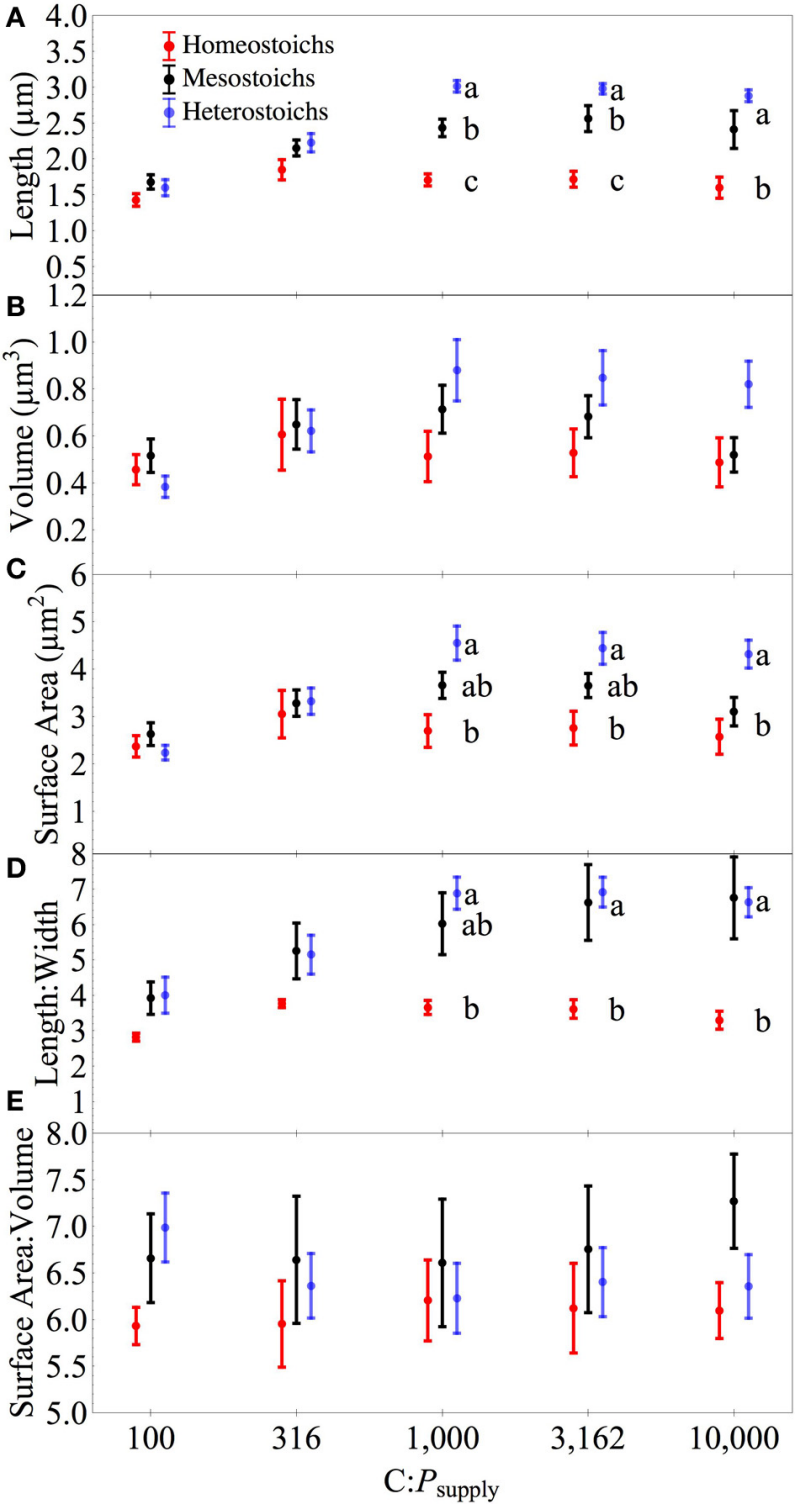
**Morphometry of isolates by stoichiometric category**. Cell morphometry of the isolates across C:P_supply_. Error bars denote one standard error of the mean. Panels for length **(A)**, volume **(B)**, surface area **(C)**, length: width **(D)**, and surface area: volume **(E)**. Lower case letters denote significantly different subsets of the categories at each level of C:P_supply_ (Tukey HSD, *p* < 0.05).

The isolates exhibited a range of morphological responses to P limitation (Figure 8) and these responses were related to stoichiometric category. At high C:P_supply_, homeostoich strains increased less in length, volume, surface area, and L:W than the mesostoich and heterostoich strains (Supplement Figures S1–S3). Mean cell length was significantly affected by stoichiometric category, C:P_supply_, and an interaction (all *p* < 0.0001). Cell L:W was affected by category (*p* < 0.0001) and C:P_supply_ (*p* < 0.0001) without a significant interaction (*p* > 0.05). Eighteen strains exhibited significantly increased length:width (L:W) under P limitation (Supplement Figures S1–S3). Overall, cell SA:V did not show significant effects of category or C:P_supply_. Eight homeostoich and mesostoich strains exhibited significantly increased surface area:volume (SA:V) in response to P limitation (2–25% change). In contrast, all but one of the heterostoich strains showed a significant decrease in SA:V under P limitation. Cell volume showed significant effects of stoichiometric category, C:P_supply_, and an interaction (all *p* < 0.05), again with heterostoichs increasing most in volume under P limitation. Cell surface area showed significant effects of category, C:P_supply_, and an interaction (all *p* < 0.001).

## Discussion

The strains examined in this study showed more variability in their elemental content and stoichiometric regulation than was previously known for heterotrophic bacteria. Data from these experiments can be used to provide insight into three areas. First, these cultures illuminate a gradient of stoichiometric strategies and show that isolates from a single assemblage may exhibit strong homeostasis or flexible stoichiometry depending upon the isolation conditions used. Second, heterostoich strains achieved flexibility in their stoichiometry through low P quotas at all P supply levels and dynamic C content, whereas homeostatic strains had high quotas of both C and P. Last, cell morphology and stoichiometric flexibility are related in most strains, but existing hypotheses receive only partial support from the data presented here.

### Isolation conditions select for stoichiometric regulation

The medium formulations employed in this study differed in their effectiveness for culturing strains from the bacterial assemblages in Lake Itasca and Long Lake (MN). Notably, more strains were viable in the BMM formulations than in the nutrient broth medium. Previous studies have demonstrated that only a small portion of the bacterial community can be easily cultured (Staley and Konopka, 1985; Eilers et al., 2000) and that nutrient-rich media are often poorly suited to isolate bacteria from aquatic environments (Barer and Harwood, 1999). For the defined medium dilution cultures, there was a decrease in apparent cultivability when the P availability was decreased by three orders of magnitude. Thus, there were distinct effects of C source (large effect) and P availability (smaller effect) on the number of culturable cells. Fewer bacteria were capable of utilizing the animal-derived carbon substrates in the nutrient broth compared to the defined medium, which could be attributed to inhibition of growth in some strains by high concentrations of substrates (Morita, 1997).

The medium formulations used to isolate strains from Long Lake and Lake Itasca effectively selected for strains with different types of stoichiometric regulation. Both the P-rich and P-poor media formulations yielded isolates exhibiting a range of stoichiometric regulation, but the P-rich media produced disproportionately more homeostatic strains compared to P-poor media. Previous studies on the stoichiometry of bacterial isolates have examined only strains that were isolated on nutrient-rich media (Nakano, 1994; Løvdal et al., 2008; Scott et al., 2012). Although some isolates obtained using nutrient-rich media have exhibited non-homeostasis and high C:P_biomass_ (Scott et al., 2012), these studies have described a comparatively small portion of the stoichiometric strategies culturable from natural assemblages. Although our isolation methods produced a broader range of physiologies than previous work, the defined medium used for the chemostat cultures restricts the number of strains that could be cultured. Further work using other carbon substrates and strains would help to determine if the gradient of stoichiometric flexibility is representative for freshwater isolates.

### Gradient of stoichiometric regulation in isolates

The isolates described here showed a substantial range of stoichiometric regulation, from strong homeostasis to highly flexible stoichiometry. This finding unequivocally demonstrates that bacteria differ in their strength of stoichiometric regulation and that assemblages contain multiple stoichiometric strategies. The first systematic examination of stoichiometric homeostasis in heterotrophic bacteria was performed with *E. coli*, which was strongly homeostatic (Makino et al., 2003). Other studies did not find such strong homeostasis as in *E. coli*, with most strains exhibiting only weak or moderate homeostasis (Chrzanowski and Kyle, 1996; Løvdal et al., 2008 Scott et al., 2012). From these experiments, it appears that *E. coli* could represent an aberrant observation due to its high absolute growth rate relative to other bacteria (dilution rates of 0.5–1.5 h^−1^) or due the culture conditions used. However, the homeostoich strains characterized here exhibited modest growth rates that did not differ from the mesostoich or heterostoich strains, suggesting that strong homeostasis is not simply a signature of a high growth rate, either absolute growth rate (μ_max_) or realized growth rate. This is contrary to our prediction, but the BMM formulations used in this study do not reflect the maximum growth rate of the strains *in situ*. Instead, the existence of strongly homeostatic strains could represent physiological adaptation to environments with high nutrient availability and low imbalance. Furthermore, homeostatic strains of bacteria can be dominant at low C:P_supply_ (Godwin and Cotner, 2014), but heterostoich physiology is dominant at high C:P_supply_, suggesting that homeostatic strains are poorly adapted to dealing with resource imbalance.

The range of stoichiometric regulation present within these isolates (slopes from 0 to 0.93) is equivalent to the range of stoichiometric regulation measured in all previously published studies of bacterial isolates and assemblages. Furthermore, this range is comparable to the extent of stoichiometric flexibility associated with species of phytoplankton (Persson et al., 2010 and references therein). Although it is often assumed that all heterotrophic bacteria are strongly homeostatic (Tambi et al., 2009; Tanaka et al., 2009; Fanin et al., 2013), this study and other recent studies with environmental isolates demonstrate that non-homeostasis is common among culturable bacteria. The range of C:N:P_biomass_ exhibited by the cultures was comparable to the range observed in assemblages of bacteria cultured from lakes (Godwin and Cotner, 2014). Taken together, these results indicate that assemblages of bacteria likely contain strains with a range of stoichiometric regulation and suggest a flexible and diverse role in carbon and nutrient cycling. The wide range of responses by mesostoich strains (Figures 6, 8) could be attributable to the arbitrary cutoff values used to assign stoichiometric categories. Further work with a large number of strains from different environments would help to determine whether distinct subgroups exist with assemblages or there is a continuous gradient of stoichiometric regulation.

### Stoichiometry and cell quotas

Although the strains differed in the extent of plasticity in their C and P quotas, several key patterns are apparent at the level of the stoichiometric categories. The first pattern is that although the isolates had similar C:N:P_biomass_ under P-replete conditions, heterostoichs as a group had lower P content than the other categories at all levels of C:P_supply_. This pattern of limited variation in C:P_biomass_ under P sufficiency (Scott et al., 2012) suggests that heterotrophic bacteria have an essential C:P_biomass_ under conditions of low imbalance. This is similar to the convergence of phytoplankton species at N:P_biomass_ of approximately 16:1 under P-sufficiency (Hillebrand et al., 2013). Heterostoichs achieved plasticity in C:P_biomass_ by the combination of a uniformly low cellular P content and accumulated C under P-stress. The C and P quotas of the homeostoich strains were higher and changed less compared to the heterostoichs. No single measure is sufficient to definitively diagnose P nutritional status or resource imbalance. In particular, neither C:P_biomass_ nor P quotas could be used to reliably diagnose P limitation in homeostoich strains. Instead, alternative measures such as transcriptional profiling (Boer et al., 2010), phosphatase activity (Cotner and Wetzel, 1991), or growth rate bioassays (Cotner et al., 1997; Sterner et al., 2004) would be more informative.

Since the P content of the heterostoichs was lower than the other categories even at high P availability, it seems likely that a lower overall P quota is required for highly flexible biomass stoichiometry. Reduced P content and flexible C quotas of the heterostoich strains can also explain higher cell abundance and higher apparent yields of C and P. The Growth Rate Hypothesis (GRH) predicts that the P content of an organism is proportional to its growth rate due to the role of P-rich ribosomes in growth (Elser et al., 2000). The strength of stoichiometric homeostasis was not correlated with maximum growth rate, but the P content of the isolates under P sufficient conditions was positively correlated with maximum growth rate (*r*^2^ = 0.37, *p* < 0.003). This can be explained by high variability in P content among the mesostoichs strains. From the available genome sequence data, several of the mesostoich strains are predicted to exhibit fast growth rates and exploit temporally or spatially variable resource conditions (Livermore et al., 2014). Thus, their stoichiometry under steady state chemostat culture might not represent their response to variable C and P *in situ* within aquatic ecosystems.

The second key result is that several strains increased their C content under P limitation, contributing to the elevated C:P_biomass_. This shows that the heterostoichs achieved stoichiometric flexibility by maintaining low cellular P content and increasing cellular C under P limitation and suggests that heterostoichs could alter their biomass stoichiometry via accumulation of C-rich molecules (e.g., glucose, glycogen, extracellular polymers). However, most of the isolates had relatively constrained C:N_biomass_ compared to C:P_biomass_ or N:P_biomass_, which suggests that any macromolecules used for surplus storage of C also contained a substantial amount of N. One exception to this pattern is strains D301 and D304, which accumulated biomass with high C:N (43–44), characteristic of accumulation of a C-rich material such as poly-B-hydroxybutyrate or glycogen. Part of the measured increase in C:P_biomass_ is attributable to an increase in cell volume (see below).

The third key result is that in addition to lower P content and variable stoichiometry, the heterostoich strains had higher apparent C and P yields than mesostoich and homeostoich strains, regardless of the C:P_supply_. The higher apparent C yields for the heterostoichs could be explained by high carbon use efficiency (Sinsabaugh et al., 2013) or surplus uptake of C when P limited. Assuming that the heterostoichs were C limited at 100:1 and consumed all of the available glucose, the apparent yields represent a carbon use efficiency of approximately 30%. The apparent P yield at C:P_supply_ of 100:1 was low across categories, reflecting P sufficiency and incomplete consumption of P. At intermediate levels of C:P_supply_, the heterostoichs utilized more of the available P due to low P quotas. Apparent P yields for homeostoichs and mesostoichs increased with increasing C:P_supply_, but the heterostoich apparent P yield decreased. As the input of P decreases, the steady state residual P becomes large relative to the assimilated portion of the available P, making the apparent P yield lower. Together with the observation that many of the heterostoichs had regression breakpoints (C:P_TER_, Sterner and Elser, 2002) of 1000:1 or greater (Figure 3), this pattern indicates that the heterostoichs became P-limited at higher C:P_supply_ than the mesostoichs or homeostoichs. An increased C:P_TER_ supports the hypothesis that heterostoichs have superior competitive ability at intermediate and high C:P_supply_.

### Role of morphometry in stoichiometry and quotas

Thingstad et al. (2005) documented an increase in cell length to width ratios under P limitation and hypothesized that this is an adaptation to increase the surface area for uptake of P across the cell membrane. This hypothesis is partially supported by the present study: the mesostoich and heterostoich strains increased their surface area but there was no change relative to their cellular volume. The allometric scaling of cell size and surface area is dependent upon cell shape and also the absolute dimensions of the cells (Grover et al., 2004; Okie, 2013). At the dimensions of these cells, increasing length without changing width increases the surface area of the cell, but also increases volume, leading to little change in SA:V. In contrast, decreases in cell width would lead to increased L:W and also increased SA:V. Although morphometric elongation was a common response to P limitation among these isolates, there was an important difference between the stoichiometric categories. Most of the homeostoich and mesostoich strains increased both their L:W and SA:V slightly under P limitation and accumulated biomass with a modest C:P ratio. For heterostoichs, the morphological change resulted in tight coupling of surface area and volume. Since the heterostoich strains increased their surface area under P limitation (but decreased SA:V) by adding biomass that was deplete in P, the increase in cellular volume might not represent a significant cost, supporting the hypothesis that surplus C can be used to increase diffusive uptake of P Thingstad et al. (2005).

### Implications

This study highlights the importance of physiological constraints in biomass stoichiometry. Bacteria in aquatic ecosystems are commonly assumed to have low C:P_biomass_, high P content, and little flexibility in their elemental composition, but these assumptions are challenged by the physiology of the isolates presented here and other recent work. Compared to an assemblage consisting of only homeostatic high-P bacteria, an assemblage composed of multiple stoichiometric strategies should be more sensitive to changes in the availability of C and P. Bacteria with flexible biomass stoichiometry can buffer changes in ambient C and P by altering their biomass composition. If stoichiometric flexibility is linked to competitive ability for P (through minimum P quotas), anthropogenic inputs of inorganic P may lead to decreased abundance of flexible strains and would serve to decouple the consumption of organic C and uptake of inorganic P. Such assemblage-level dynamics are important to understanding how bacteria link multiple element cycles and underscore the need to describe functional diversity of strains present within ecosystems.

Although the bacterial strains described in the present study were isolated using a range of culture methods, these strains do not necessarily represent the physiology of the dominant taxa in freshwater ecosystems. Also, all of the physiological measurements were obtained using a defined medium with a single carbon substrate. This could cause overestimation of stoichiometric flexibility since the bacteria experienced strong resource imbalance at high C:P_supply_. While all of the isolates genera have been detected in lakes using 16S sequencing (Newton et al., 2011), their global representation within lake assemblages is not known. The isolate Pnec (*Polynucleobacter necessarius*) is a comparatively well-studied representative from lakes (Livermore et al., 2014), often representing a large fraction of bacterial assemblages (Jezberova et al., 2010; Hahn et al., 2012). Because Pnec is ubiquitous in lakes and was moderate in its biomass stoichiometry and growth rate, it could serve as a model strain for bridging culture-based physiological information with sequence-based characterization of *in situ* assemblages. None of the isolates exhibited homeostasis at high C:P_biomass_ or N:P_biomass_, but single-cell measurements of bacterial element content from lakes show significant variation in C:P_biomass_ and N:P_biomass_(Fagerbakke et al., 1996; Cotner et al., 2010). Together, these findings suggest that most cells present *in situ* have flexible stoichiometry and experience elemental imbalance. Assessing the relative abundance of homeostatic and flexible strains within lakes is essential for the development of assemblage-scale models with stoichiometric constraints, particularly where assemblages are subject to strong resource imbalance.

### Conflict of interest statement

The authors declare that the research was conducted in the absence of any commercial or financial relationships that could be construed as a potential conflict of interest.
