# Supplementary material for: Stoichiometric flexibility in diverse aquatic heterotrophic bacteria is coupled to differences in cellular phosphorus quotas
Source: Front Microbiol. 2015 Feb 27;6:159. doi: 10.3389/fmicb.2015.00159 (PMC4343017; doi:10.3389/fmicb.2015.00159)
Supplement: Supplementary file 1 [file DataSheet1.DOCX]

Cell Morphometry Calculations

Equations for a cylinder capped with two hemispheres were adapted from Hillebrand et al (1999).

Surface area given length (*l*) and width (*w*):

$$SA=l\cdot w\cdot\pi$$

Volume (*V*) given length and width:

$$V=\frac{1}{12}\left( 3\cdot l-w \right)\cdot w^{2}\cdot\pi$$

Surface area : volume (*SA:V*) given length and width:

$$SA:V=\frac{12\cdot l}{3\cdot l\cdot w-w^{2}}$$

Length given planar area (*s*) and perimeter (*p*):

$$\frac{2\cdot p+\left( \pi-2 \right)\cdot\sqrt{p^{2}-4\cdot\pi\cdot s}}{2\cdot\pi}$$

Width given planar area and perimeter:

$$w=\frac{p-\sqrt{p^{2}-4\cdot\pi\cdot s}}{\pi}$$

Volume given planar area and perimeter:

$$v=\frac{\left( p-\sqrt{p^{2}-4\cdot\pi\cdot s} \right)^{2}\cdot\left( 4\cdot p+\left( 3\cdot\pi-4 \right)\cdot\sqrt{p^{2}-4\cdot\pi\cdot s} \right)}{24\cdot\pi^{2}}$$

Surface area given planar area and perimeter:

$$SA=\frac{-p^{2}\cdot\left( \pi-4 \right)+4\cdot\pi\cdot s\cdot\left( \pi-2 \right)+p\cdot\left( \pi-4 \right)\cdot\sqrt{p^{2}-4\cdot\pi\cdot s}}{2\cdot\pi}$$

Length : width ratio given planar area and perimeter:

$$L:W=\frac{-4\cdot s\cdot\left( \pi-2 \right)+p\cdot\left( p+\sqrt{p^{2}-4\cdot\pi\cdot s} \right)}{8\cdot s}$$

Surface area : volume ratio given planar area and perimeter:

$$SA:V=\frac{12\cdot\pi\cdot\left( 2\cdot p+\left( \pi-2 \right)\cdot\sqrt{p^{2}-4\cdot\pi\cdot s} \right)}{\left( -p+\sqrt{p^{2}-4\cdot\pi\cdot s} \right)\cdot\left( 4\cdot p+\left( 3\cdot\pi-4 \right)\cdot\sqrt{p^{2}-4\cdot\pi\cdot s} \right)}$$

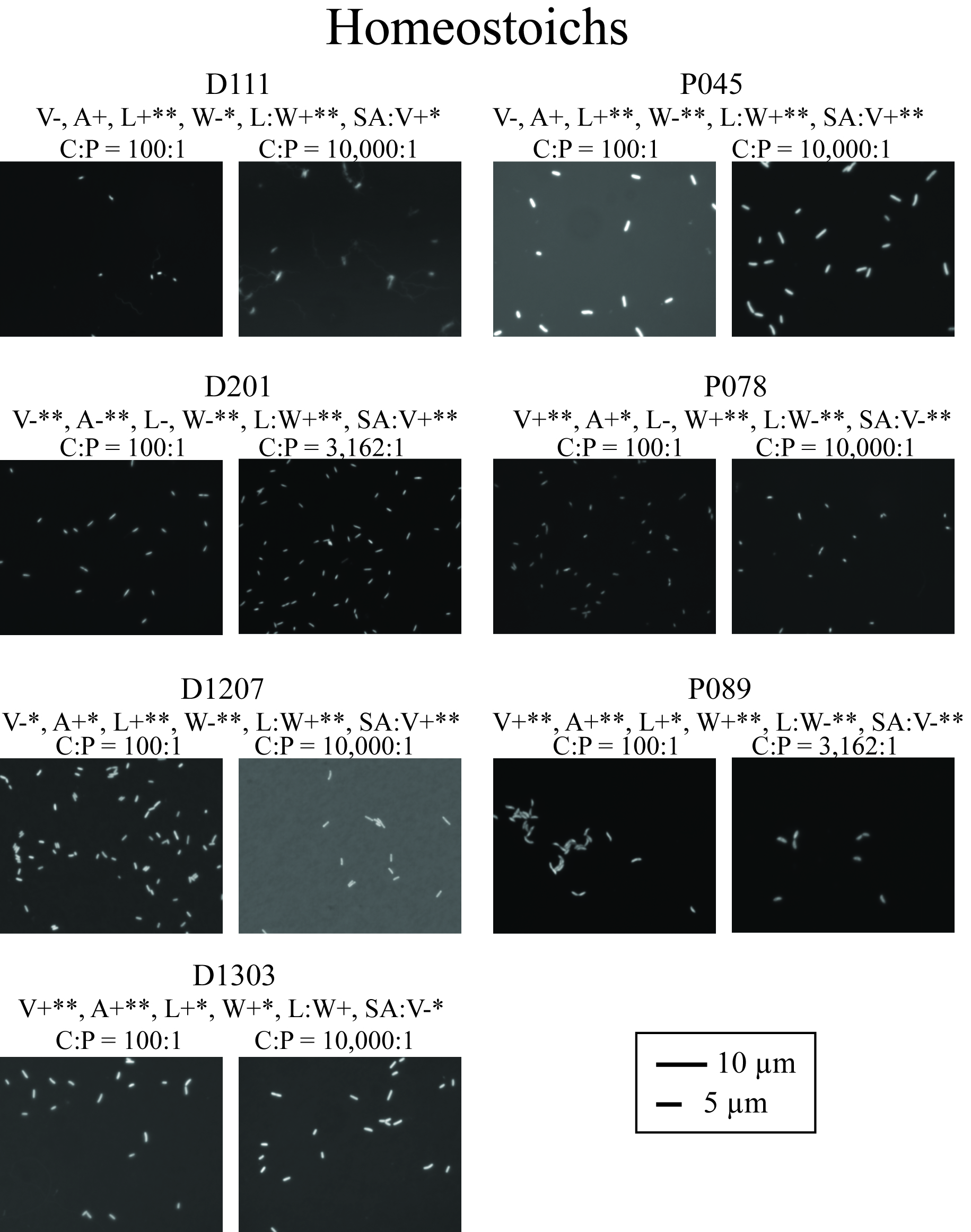


Supplement Figure S1. Photomicrographs of homeostoich strains from chemostats at high and low C:P_supply_. For each strain, the absolute direction of changes in morphometry under P limitation are given for volume (V), area (A), length (L), width (W), length : width (L:W), and surface area : volume (SA:V), with significance in ANOVA denoted as p < 0.05 (*) and p < 0.0001 (**). Scale bars are for all images.


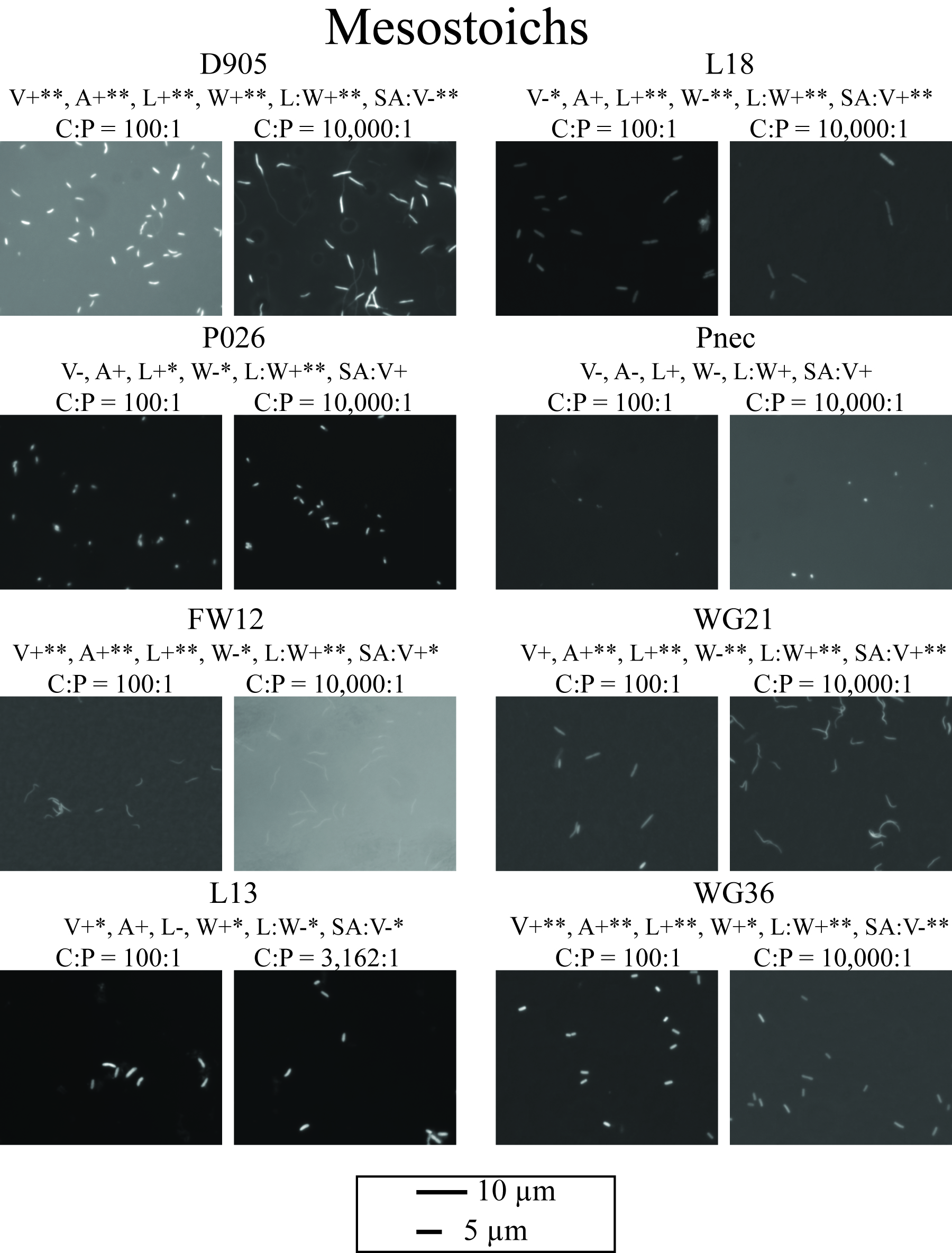


Supplement Figure S2. Photomicrographs of mesostoich strains from chemostats at high and low C:P_supply_. For each strain, the absolute direction of changes in morphometry under P limitation are given for volume (V), area (A), length (L), width (W), length : width (L:W), and surface area : volume (SA:V), with significance in ANOVA denoted as p < 0.05 (*) and p < 0.0001 (**). Scale bars are for all images.


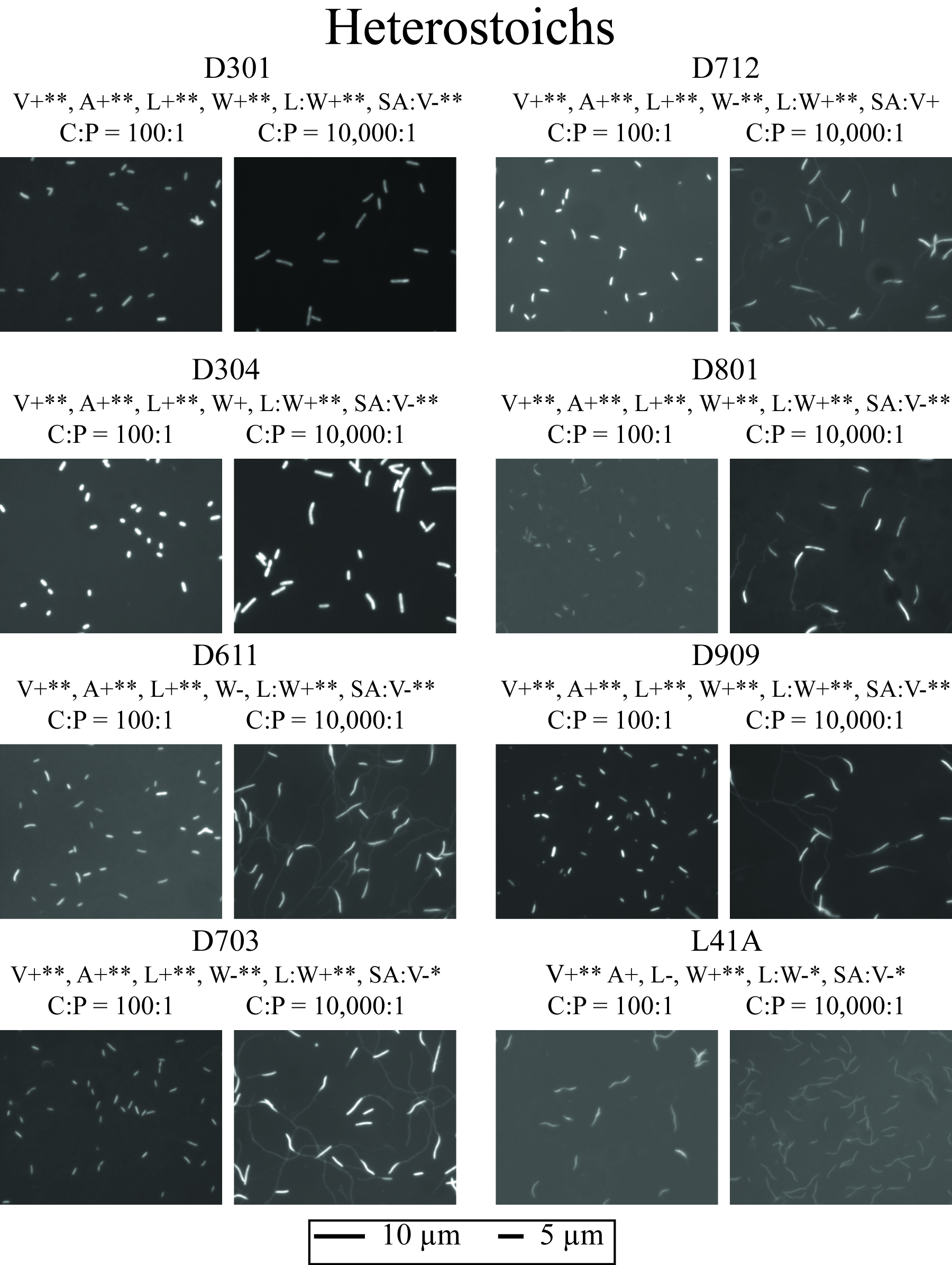


Supplement Figure S3. Photomicrographs of heterostoich strains from chemostats at high and low C:P_supply_. For each strain, the absolute direction of changes in morphometry under P limitation are given for volume (V), area (A), length (L), width (W), length : width (L:W), and surface area : volume (SA:V), with significance in ANOVA denoted as p < 0.05 (*) and p < 0.0001 (**). Scale bars are for all images.
